# RETRACTION: Risk Assessment and Pathogen Profile of Surgical Site Infections in Traumatic Brain Injury Patients Undergoing Emergency Craniotomy: A Retrospective Study

**DOI:** 10.1111/iwj.70658

**Published:** 2025-04-23

## Abstract

**RETRACTION:** FengW. , SunC. , YangS. Hao J. , WangP. , WangZ. , LiuX. , LouJ. , and YangY. , “Risk Assessment and Pathogen Profile of Surgical Site Infections in Traumatic Brain Injury Patients Undergoing Emergency Craniotomy: A Retrospective Study,” International Wound Journal 21, no. 3 (2024): e14743, 10.1111/iwj.14743.38420721 PMC10902686

The above article, published online on 29 February 2024, in Wiley Online Library (http://onlinelibrary.wiley.com/), has been retracted by agreement between the journal Editor in Chief, Professor Keith Harding; and John Wiley & Sons Ltd. Following an investigation by the publisher, all parties have concluded that this article was accepted solely on the basis of a compromised peer review process. The editors have therefore decided to retract the article. The authors did not respond to our notice regarding the retraction.

**RETRACTION:** W. Feng , C. Sun , S. Hao J. Yang , P. Wang , Z. Wang , X. Liu , J. Lou , and Y. Yang , “Risk Assessment and Pathogen Profile of Surgical Site Infections in Traumatic Brain Injury Patients Undergoing Emergency Craniotomy: A Retrospective Study,” International Wound Journal 21, no. 3 (2024): e14743, 10.1111/iwj.14743.38420721 PMC10902686

The above article, published online on 29 February 2024, in Wiley Online Library (http://onlinelibrary.wiley.com/), has been retracted by agreement between the journal Editor in Chief, Professor Keith Harding; and John Wiley & Sons Ltd. Following an investigation by the publisher, all parties have concluded that this article was accepted solely on the basis of a compromised peer review process. The editors have therefore decided to retract the article. The authors did not respond to our notice regarding the retraction.

